# Transient Febrile reaction after Electroconvulsive Therapy : A case report in an adult man with Ultra-Resistant Schizophrenia

**DOI:** 10.1192/j.eurpsy.2024.1466

**Published:** 2024-08-27

**Authors:** K. Abdessattar, A. Hkiri, H. khiari, O. Youssef, R. Ghachem

**Affiliations:** psychiatry department Pinel, Razi Hospital, Manouba, Tunisia

## Abstract

**Introduction:**

Electroconvulsive therapy (ECT) is a therapeutic method that induces artificial seizure by electrical stimulation to resolve various psychiatric symptoms. ECT is particularly effective in resistant schizophrenia and may improve response to medication despite the presence of potential adverse side effects. Post-ECT delirium and Headaches are some of the most frequent side effects presented in literature. Fever is yet another unexplained reaction, however there are a few case reports and retrospective studies that report on it.

**Objectives:**

We aim to illustrate through a clinical case and a review of literature the prevalence of post ECT fever as well as the possible explanatory mechanisms.

**Methods:**

In this study we report the case of a man with ultra-resistant schizophrenia who was treated successfully with ECT despite the development of transient febrile reaction and we present a review of literature on pubmed using the following key words : ECT, fever,resistant psychosis, mechanisms.

**Results:**

Our patient is a 48-year-old man with a psychiatric history of schizophrenia evolving since the age of 34. He has a history of matricide in 2021 resulting in his hospitalisation in a forensic psychiatric ward. He underwent trials of classic and atypical antipsychotics that weren’t efficacious thus he was diagnosed with resistant schizophrenia in 2022. He was treated initially with clozapine 500 mg per day and then with the association (clozapine + amisulpride) yet it wasn’t effective on his persecutory delirium and fratricide ideas. Plus, there was no reduction in his PANSS (Positive and Negative Syndrome Scale) scores. The diagnosis of ultra-resistant schizophrenia was established. The staff indicated the adjunction of ECT to Clozapine. In the inpatient unit, hours after his fourth ECT session he developed a fever (40°C), his blood pressure (120/80 mm Hg), pulse(85 beats per minute),and respiratory rate(20 breaths per minute) were normal. Blood samples, including cultures, were drawn, which showed normal blood cell count and CPK(140U/L) but CRP was elevated (31 U/L), a chest x-ray showed no acute pulmonary disease, and his urinalysis result and Covid test were negative. His fever resolved then spontaneously after two hours. The same transient febrile reaction occurred again 3 times. It was postulated in literature that fever may be due to inadequate muscle reaction. Data also suggested the potential influence of ECT on the hypothalamus that is a key region in regulating body temperature.

**Conclusions:**

Further studies are required in order to establish the real prevalence of this side effect and its possible causes.

**Disclosure of Interest:**

None Declared

